# Case report: successful closure of a large macular hole secondary to uveitis using the inverted internal limiting membrane flap technique

**DOI:** 10.1186/s12886-015-0072-5

**Published:** 2015-07-25

**Authors:** Masayuki Hirano, Yuki Morizane, Tetsuhiro Kawata, Shuhei Kimura, Mio Hosokawa, Yusuke Shiode, Shinichiro Doi, Mika Hosogi, Atsushi Fujiwara, Fumio Shiraga

**Affiliations:** Department of Ophthalmology, Okayama University Graduate School of Medicine, Dentistry and Pharmaceutical Sciences, 2-5-1 Shikata-cho Kita-ku, Okayama City, Okayama 700-8558 Japan

**Keywords:** Macular hole, Uveitis, Inverted internal limiting membrane flap technique

## Abstract

**Background:**

Macular holes (MHs) are one of the complications of posterior uveitis that can significantly disturb vision. Conventional MH surgery (vitrectomy, internal limiting membrane (ILM) peeling, and gas tamponade) has been reported to show lower closure rates in patients with MHs secondary to uveitis than in patients with idiopathic MHs. Recently, the inverted ILM flap technique has been reported to be effective for treating refractory MHs. Here, we describe the application of this technique in a patient with a large MH secondary to uveitis, and its successful closure.

**Case presentation:**

An 80-year-old woman presented with a chronic, large MH secondary to uveitis. The minimum aperture diameter of the MH was 569 μm and extensive post-inflammatory chorioretinal atrophy was present, which included the juxtafoveal region. Vitrectomy with the inverted ILM flap technique assisted by low molecular weight hyaluronic acid was performed. Three days after surgery, the MH was closed successfully, without excessive gliosis.

**Conclusion:**

The inverted ILM flap technique may be the preferred surgical procedure for the treatment of large MHs secondary to uveitis.

## Background

Macular holes (MHs) are one of the complications in uveitis that can significantly affect sight. Conventional MH surgery (i.e. vitrectomy, ILM removal, and gas tamponade) has been reported to show a low closure rate for MHs secondary to uveitis [1] compared to idiopathic MH [2, 3], because MHs tend to become larger in diameter, with the retina showing reduced extensibility, after retinal inflammation. However, the most effective surgical procedure for MHs secondary to uveitis has yet to be established.

Several recent studies have reported the efficacy of the inverted ILM flap technique for treating refractory MHs [4, 5]. Michalewska *et al.* have reported that using the inverted ILM flap technique prevented MHs having a flat-open appearance after surgery and improved both the closure rate (98 %) and the postoperative visual acuity for MHs with a diameter greater than 400 μm [4]. Kuriyama *et al.* have also reported a closure rate of 80 % for myopic MHs treated using this procedure [5].

In this case report, we describe the application of the inverted ILM flap technique to the treatment of a large MH secondary to uveitis.

## Case presentation

### Presentation, history, and ocular examination

**Fig. 1:**
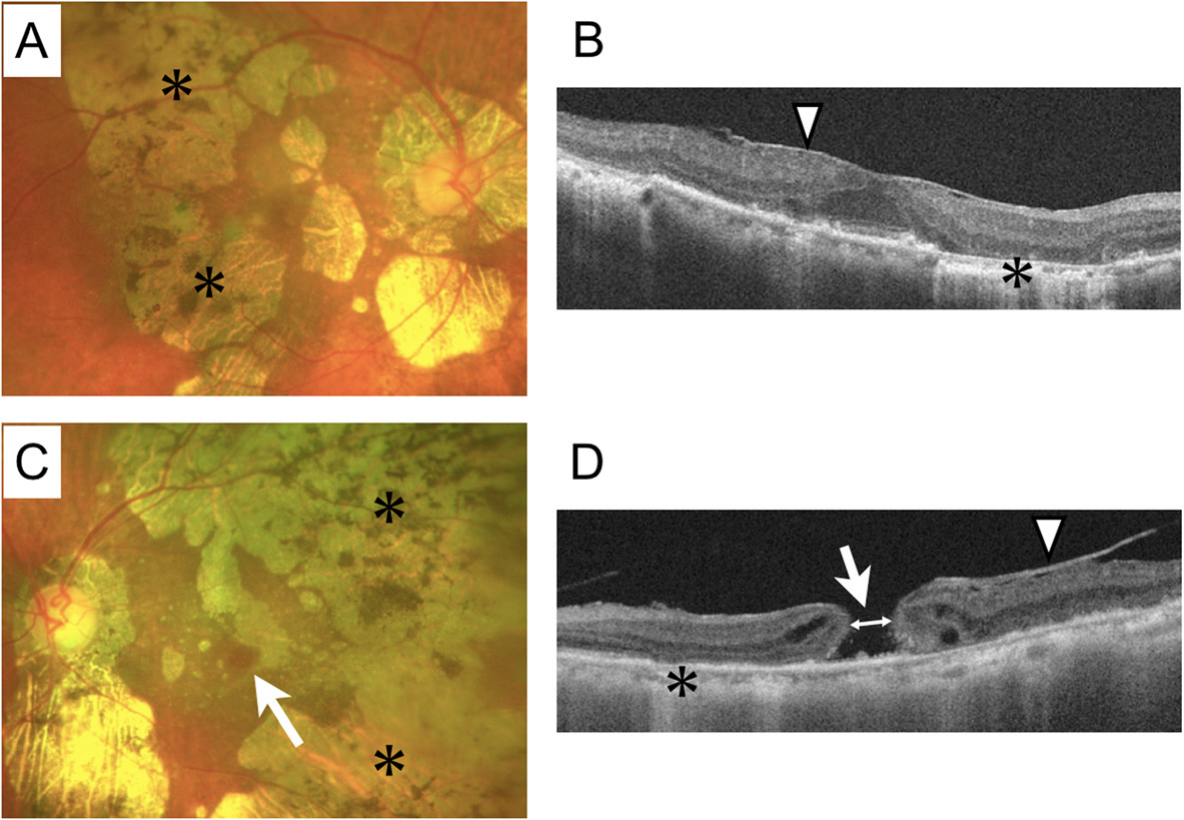
Preoperative fundus examination and swept source optical coherence tomography of the eyes of the 80-year-old female patient. **a** Color photograph of the fundus of the right eye (best corrected visual acuity = 20/40) showing patchy chorioretinal atrophy and retinal degeneration (asterisk) extending from the posterior pole to the midperiphery including the juxtafoveal region. **b** Swept source optical coherence tomography (SS-OCT) image of the right eye showing epiretinal membrane formation (arrow head), thinning of the retina and choroid corresponding to chorioretinal atrophy and retinal degeneration (asterisk), ellipsoid zone defect, and irregularity in the pigment epithelium. **c** Color photograph of the fundus of the left eye (best corrected visual acuity = 20/285) showing a full-thickness macular hole (arrow), in addition to the features seen in the right eye (**a**). **d** SS-OCT image of the left eye showing the full-thickness large macular hole (arrow), which was 569 μm in diameter (double-headed arrow shows where we measured the minimum aperture diameter), in addition to the features seen in the right eye (**b**) (arrow head shows epiretinal membrane formation)

An 80-year-old female was referred to our clinic, mainly complaining of a loss of visual acuity in her left eye, which had lasted more than 4 months. She had a history of bilateral uveitis of unknown cause, but the inflammation had not been active for more than 10 years. The patient had no significant history of systemic disease. At her initial visit, the best corrected visual acuity (BCVA) was 20/40 in her right eye and 20/285 in her left eye. The anterior segments of both her pseudophakic eyes were normal and no inflammatory cells were observed. A fundus examination showed slightly pale optic discs in both eyes and a full-thickness MH in the left eye (Fig. 1). In both eyes, patchy chorioretinal atrophy and retinal degeneration with hyperpigmentation extended from the posterior pole to the mid-periphery including the juxtafoveal region.

### OCT findings

Swept source optical coherence tomography (SS-OCT; DRI OCT-1 Atlantis, TOPCON Corporation, Tokyo, Japan) confirmed the presence of a full-thickness MH, with a minimum aperture diameter of 569 μm, in the left eye (Fig. 1d). Epiretinal membrane (ERM) formation, the thinning of the retina and choroid corresponding to chorioretinal atrophy and retinal degeneration, ellipsoid zone defects, and irregularities in the retinal pigment epithelium (RPE) were observed in both eyes. The central retinal thickness was 276 μm in the right eye and the MH height was 244 μm in the left eye.

### Surgical procedure

**Fig. 2:**
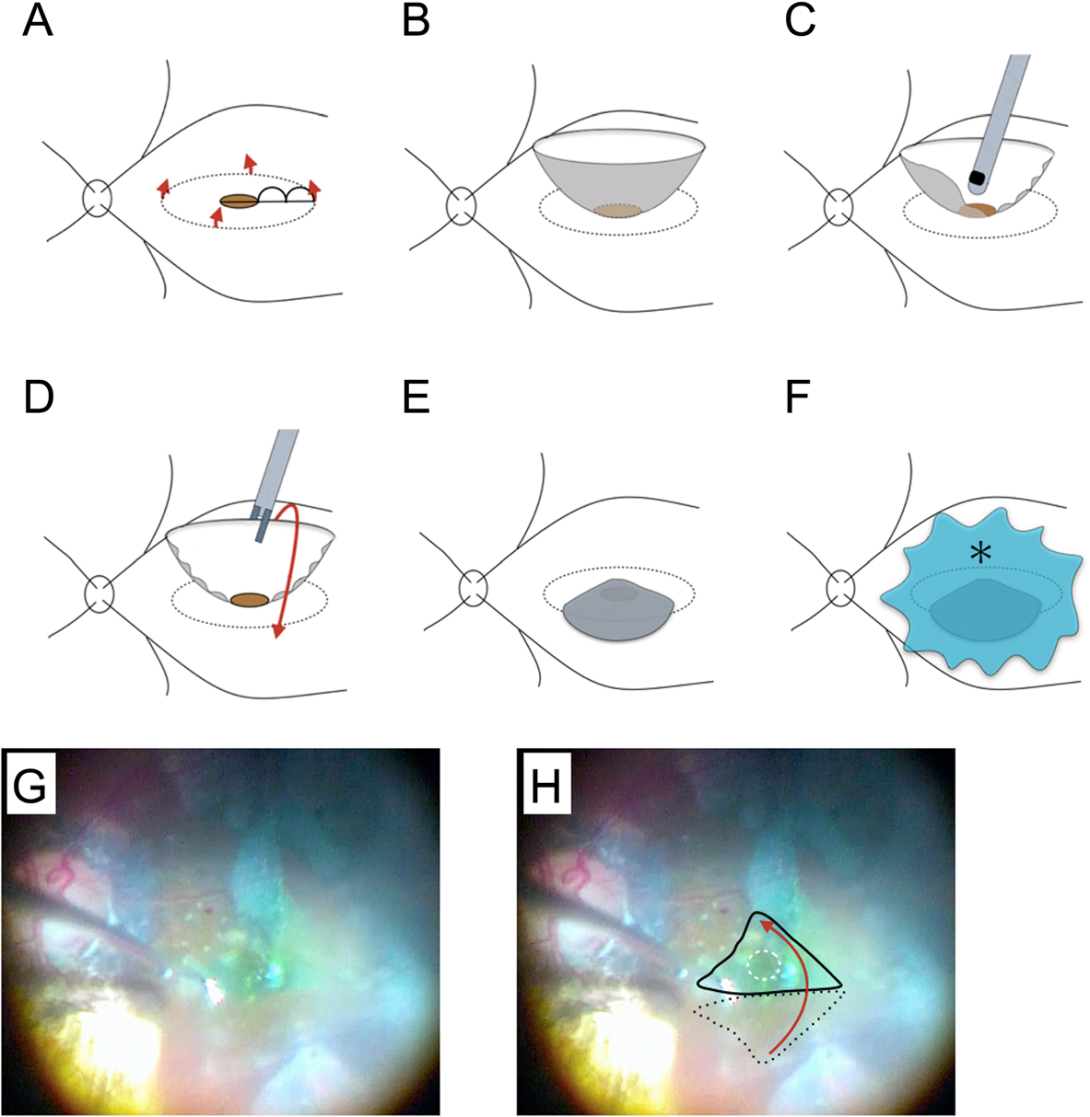
Diagrammatic representation of the modified inverted internal limiting membrane (ILM) flap technique and intraoperative photographs of the patient’s left eye. **a** After performing a 25-gauge micro-incision vitrectomy, the ILM was peeled off in a circular fashion. Red arrows show the direction of ILM peeling. The radius of the area of ILM peeled off was approximately 2.5 times the diameter of the macular hole (MH). **b** The ILM was not removed completely from the retina and remained attached to the edge of the MH. **c** We then trimmed half of the peeled ILM using a vitreous cutter, to facilitate the inversion of the ILM. **d, e** The remnant ILM was inverted with intraocular forceps to cover the entire MH. **f** The inverted ILM flap was covered with 1 % low molecular weight hyaluronic acid (asterisk) to stabilize it. Finally, we performed fluid-air exchange, but left the hyaluronic acid in the eye. **g** Intraoperative photograph showing the inverted ILM flap. **h** Explanatory drawing of (**g**), showing the area of the ILM flap before inversion (black dotted line), how we inverted the ILM flap (red arrow), the entire MH (white dotted line), and how we covered it with the inverted ILM flap (solid black line)

Since the SS-OCT results described above indicated that stretching the retina sufficiently to close the MH would be difficult, we decided to perform a vitrectomy using the inverted ILM flap technique, based on the method reported by Michalewska *et al.* [4], but with slight modifications (Fig. 2). After performing a 25-gauge micro-incision vitrectomy, using intraocular forceps, the ILM was peeled away from the periphery towards the MH in 4 directions (Fig. 2a), but was not completely detached from the retina, leaving the attachment to the edge of the MH in place, so that a bowl-shaped ILM flap was created (Fig. 2b). The radius of the area of the ILM peeled away was approximately 2.5 times the diameter of the MH. We initially used Coomassie brilliant blue G250 solution (CBBG; Sigma-Aldrich, St. Louis, Missouri, USA) to visualize the ILM, but we re-stained the ILM with indocyanine green (ICG) to further improve visibility. As shown in Fig. 2c, we then trimmed half of the peeled ILM using a vitreous cutter, to facilitate the inversion of the ILM. The remnant ILM was inverted to cover the entire MH (Fig. 2d, e, g and h) and 1 % low molecular weight hyaluronic acid (Opegan; Santen Pharmaceutical Co. Ltd, Osaka, Japan) was placed over the inverted ILM flap to stabilize it. We performed fluid-air exchange, leaving the hyaluronic acid in the eye (Fig. 2f) and at the end of surgery the vitreous cavity was filled with 20 % SF_6_ gas. The patient remained face-down for 3 days after surgery.

### Post-operative recovery

**Fig. 3:**
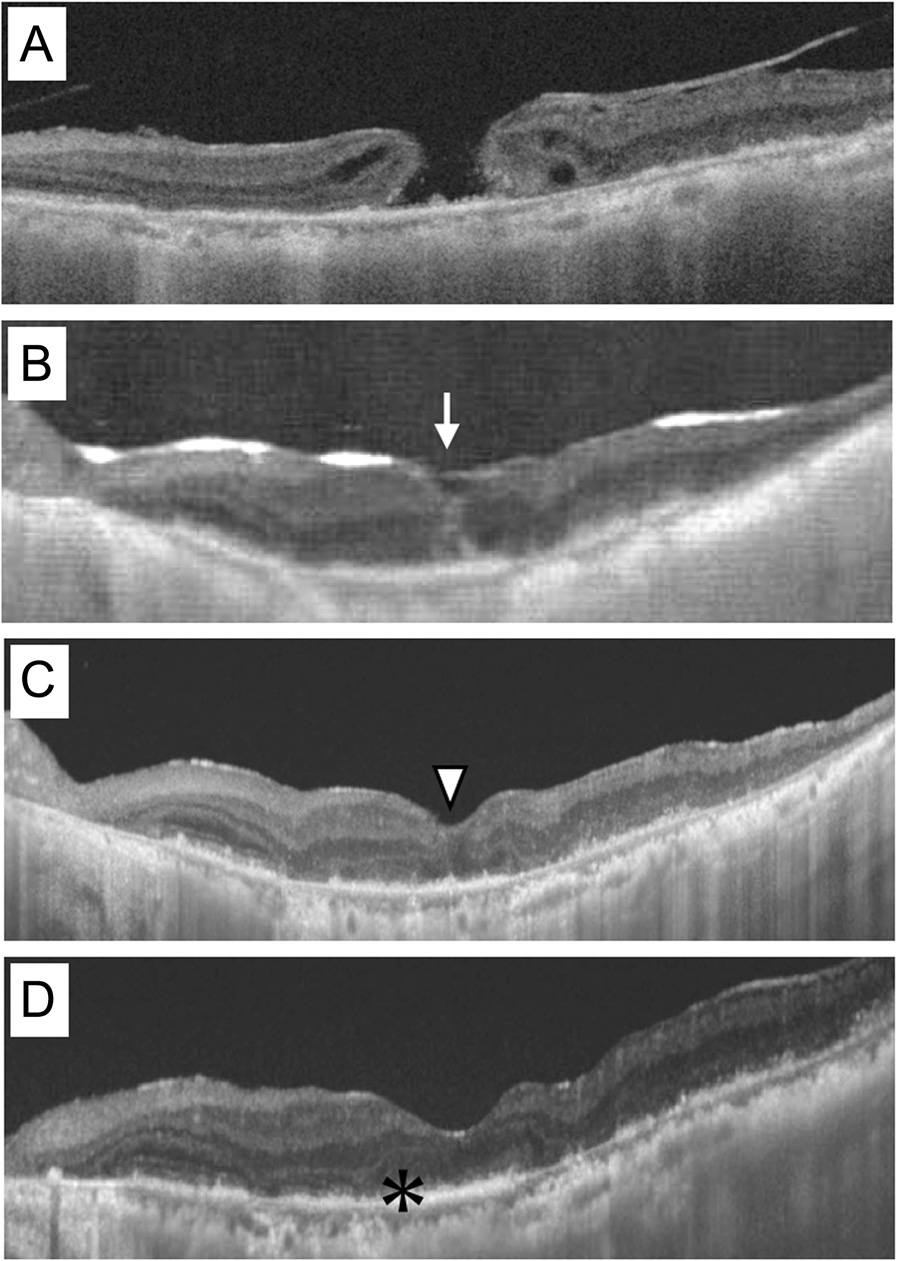
Swept source optical coherence tomography images showing the results of the inverted ILM flap technique. **a** The full-thickness large MH, which was 569 μm in diameter at the initial visit. **b** Three days after surgery, the image showed closure of the MH (arrow) in the gas-filled eye. **c** Twelve days after the surgery, the foveal contour had further improved (arrow head). **d** Although the MH had successfully closed 6 month after the surgery, leaving a U-shape depression, the ellipsoid zone remained defective (asterisk) and the visual acuity of the left eye was 20/400

Six hours after surgery, the SS-OCT image of the gas-filled eye showed that the diameter of the MH had decreased. Twenty-four hours after surgery, the borders of the MH were even closer together. Three days after surgery, the SS-OCT image showed closure of the MH (Fig. 3b) and 12 days after surgery, the foveal contour showed further improvement (Fig. 3c). Although the MH was successfully closed 6 months after surgery, leaving a U-shaped depression (Fig. 3d), the ellipsoid zone remained defective and the patient’s BCVA was 20/400.

## Conclusion

To the best of our knowledge, this is the first report of the successful closure of a large MH secondary to uveitis using the inverted ILM flap technique. Hassan *et al.* reported a closure rate of 17 % for MHs secondary to uveitis after vitrectomy, ILM peeling, and gas or silicone tamponade [1], which was much lower than the rate for idiopathic MHs [2, 3]. They suggested that the reasons for this lower closure rate was the presence of damage to the retina and retinal pigment epithelium due to inflammation and the large diameter of the MHs [1]. In addition to these observations, our case presented with ERM formation, post-inflammatory chorioretinal atrophy, and retinal degeneration including the juxtafoveal region, which can cause adhesion of the retina and RPE in this area. The presence of these conditions in the eye led us to use the inverted ILM flap technique instead of conventional ILM peel in this patient.

Although we achieved a successful closure of the MH in this patient, long-term observation will be needed to check for excessive gliosis in the retina. Michalewska *et al.* hypothesized that the inverted ILM flap technique stimulates proliferation of glial cells that fill MHs, thereby enhancing closure and improving MH closure rates [4]. In the neural tissues of the central nervous system and the retina, activation of glial cells occurs in response to any form of injury or disease and can have both protective and detrimental effects [6]. Müller cells are the principal glial cells in the retina and play of crucial roles in supporting neuronal function [7]. At the early stage of tissue damage, gliosis is an important neuroprotective event that is thought to be a cellular attempt to limit the extent of tissue damage [7]. However, the persistent activation of glial cells may contribute to tissue damage, including endothelial dysfunction and angiogenesis [8]. In eyes with a history of uveitis, larger amount of inflammatory cytokines can be secreted in response to surgery than in normal eyes. In such cases, Müller cells may be activated, proliferate excessively along the inverted ILM flap, and may induce excessive gliosis in the retina. In our patient, however, we did not see any excessive gliosis, which appears as highly reflective material in OCT [9, 10], during the 7 month follow-up period.

Recently, Shin *et al.* have reported two modifications of the original inverted ILM technique [11]. The first modification was covering the MH with a single-layered ILM flap, which may provide a more physiological structure for glial proliferation and retinal regeneration. The second modification was keeping the inverted ILM flap in position during surgery by applying perfluoro-n-octane (PFO) over the macula. However, this modified technique required creating three flaps along MH in specific order, which ware removed to create the fourth flap to be used as cover flap, which seemed technically complicated and difficult.

In addition, it took several minutes for the PFO to evaporate completely at the end of the fluid-air exchange, while continuously removing fluid from the disc. In this study, we modified their procedure for creating a single-layered ILM flap and keeping the flap in position. First, we peeled off the ILM in a circular fashion, leaving it attached at the edge of the MH, and then trimmed half of the peeled ILM with a vitreous cutter to create a single-layered ILM flap. Second, we used low molecular weight hyaluronic acid to stabilize the inverted ILM flap and left this in place at the end of the surgery. These modifications enable us to make a single-layered ILM flap and stabilize it more easily than in the previous methods described.

We initially used CBBG to stain the ILM and then re-stained it with 0.25 % ICG. Imai *et al.* reported that an expansion of RPE atrophy occurred 1 week after inverted ILM flap technique using ICG staining [12]. They assumed that the ICG-stained ILM flap provoked the RPE damage. In our patient, however, we have observed no expansion of the pre-existing chorioretinal atrophy up to 6 months after surgery. Further observations will be required to assess the safety of using ICG staining in the inverted ILM flap technique.

In conclusion, our case report suggests that the inverted ILM flap technique may be beneficial for the treatment of large MHs, secondary to uveitis. To determine the actual efficacy of this technique, further prospective studies involving a larger number of patients will be required.

## Consent

Written informed consent was obtained from the patient for publication of this case report and accompanying images. A copy of the written consent is available for review by the Editor-in-Chief of this journal.

## Abbreviations

BCVA: Best corrected visual acuity
CBBG: Coomassie brilliant blue G250
ERM: Epiretinal membrane
ICG: Indocyanine green
ILM: Internal limiting membrane
MH: Macular hole
PFO: Perfluoro-n-octane
RPE: Retinal pigment epithelium
SS-OCT: Swept source optical coherence tomography
